# Penile Anaerobic Dysbiosis as a Risk Factor for HIV Infection

**DOI:** 10.1128/mBio.00996-17

**Published:** 2017-07-25

**Authors:** Cindy M. Liu, Jessica L. Prodger, Aaron A. R. Tobian, Alison G. Abraham, Godfrey Kigozi, Bruce A. Hungate, Maliha Aziz, Fred Nalugoda, Sanjeev Sariya, David Serwadda, Rupert Kaul, Ronald H. Gray, Lance B. Price

**Affiliations:** a Department of Environmental and Occupational Health, Milken Institute School of Public Health, George Washington University, Washington, DC, USA; b Center for Microbiomics and Human Health, Division of Pathogen Genomics, Translational Genomics Research Institute, Flagstaff, Arizona, USA; c National Institute of Allergy and Infectious Diseases, National Institutes of Health, Baltimore, Maryland, USA; d Department of Epidemiology, Bloomberg School of Public Health, Johns Hopkins University, Baltimore, Maryland, USA; e Department of Pathology, School of Medicine, Johns Hopkins University, Baltimore, Maryland, USA; f Rakai Health Sciences Program, Entebbe, Uganda; g Department of Ophthalmology, School of Medicine, Johns Hopkins University, Baltimore, Maryland, USA; h Department of Biological Sciences, Center for Ecosystem Science and Society, Northern Arizona University, Flagstaff, Arizona, USA; i Department of Medicine, University of Toronto, Toronto, Canada; New York University

**Keywords:** penile microbiome, anaerobes, cytokines, foreskin inflammation, human immunodeficiency virus, susceptibility

## Abstract

Sexual transmission of HIV requires exposure to the virus and infection of activated mucosal immune cells, specifically CD4^+^ T cells or dendritic cells. The foreskin is a major site of viral entry in heterosexual transmission of HIV. Although the probability of acquiring HIV from a sexual encounter is low, the risk varies even after adjusting for known HIV risk factors. The genital microbiome may account for some of the variability in risk by interacting with the host immune system to trigger inflammatory responses that mediate the infection of mucosal immune cells. We conducted a case-control study of uncircumcised participants nested within a randomized-controlled trial of male circumcision in Rakai, Uganda. Using penile (coronal sulcus) swabs collected by study personnel at trial enrollment, we characterized the penile microbiome by sequencing and real-time PCR and cytokine levels by electrochemiluminescence assays. The absolute abundances of penile anaerobes at enrollment were associated with later risk of HIV seroconversion, with a 10-fold increase in *Prevotella*, *Dialister*, *Finegoldia*, and *Peptoniphilus* increasing the odds of HIV acquisition by 54 to 63%, after controlling for other known HIV risk factors. Increased abundances of anaerobic bacteria were also correlated with increased cytokines, including interleukin-8, which can trigger an inflammatory response that recruits susceptible immune cells, suggesting a mechanism underlying the increased risk. These same anaerobic genera can be shared between heterosexual partners and are associated with increased HIV acquisition in women, pointing to anaerobic dysbiosis in the genital microbiome and an accompanying inflammatory response as a novel, independent, and transmissible risk factor for HIV infection.

## IMPORTANCE

We found that uncircumcised men who became infected by HIV during a 2-year clinical trial had higher levels of penile anaerobes than uncircumcised men who remained HIV negative. We also found that having higher levels of penile anaerobes was also associated with higher production of immune factors that recruit HIV target cells to the foreskin, suggesting that anaerobes may modify HIV risk by triggering inflammation. These anaerobes are known to be shared by heterosexual partners and are associated with HIV risk in women. Therefore, penile anaerobes may be a sexually transmissible risk factor for HIV, and modifying the penile microbiome could potentially reduce HIV acquisition in both men and women.

## INTRODUCTION

Reducing sexual transmission is crucial to ending the HIV pandemic. Not all sexual exposures to HIV result in productive infection, and the susceptibility of individuals to infection can be heterogeneous (1). There are known risk factors for HIV, such as inconsistent condom use, higher number of sexual partners, and circumcision status, among others (2–4); however, even when these factors are combined, they do not fully explain the heterogeneity in susceptibility (5).

The foreskin is the major site of HIV exposure and viral entry in uncircumcised heterosexual men (2–4), and the interplay between the immune responses and genital microbiome at this site could be an important determinant of host HIV susceptibility. Genital inflammation promotes HIV acquisition and transmission (6, 7). In particular, increased levels of chemoattractant cytokines interleukin-8 (IL-8) and monokine induced by interferon gamma (MIG) in the penile coronal sulcus have been linked to increased density of highly HIV-susceptible CD4^+^ T cells in the underlying foreskin tissue, with consequent increased HIV risk (8).

Proinflammatory responses, including at the genital mucosa (7, 9), can be elicited by shifts in the human microbiome (9–12) and specifically, in association with dysbiosis, which is an imbalance in the microbiome that compromises health (13, 14). Thus, changes in the genital microbiome that provoke inflammation could facilitate infection by pathogens, such as HIV; this may explain how a vaginal ecological imbalance could increase HIV risk (15, 16), or how reducing penile anaerobes could decrease HIV risk (17).

Penile anaerobes decrease significantly after medical male circumcision (17), a procedure that also reduces HIV risk in men by 60% (2–4). In the current study, we tested the hypothesis that penile anaerobe abundance directly increases HIV risk by inducing proinflammatory response in the foreskin. Using a case-control study design, we compared the microbiome and cytokine levels in the penile coronal sulci in uncircumcised men who seroconverted (cases) and uncircumcised controls who remained persistently HIV seronegative (controls) during a randomized-controlled trial of medical male circumcision in Rakai, Uganda.

## RESULTS

We assessed the associations between penile anaerobic bacteria, cytokines, and HIV acquisition, using data from 182 uncircumcised men, 46 who became infected with HIV (cases) and 136 who remained uninfected (controls), in a case-control study nested within a 2-year randomized-controlled trial of male circumcision in Rakai, Uganda (2). We focused on the 10 anaerobic genera that were most greatly reduced following male circumcision (17) and that may play a functional role in the noted association between male circumcision and reduced risk of HIV acquisition. These genera composed an average 62% of the total penile bacterial load in study participants. For each anaerobic genus of interest, we estimated its absolute abundance, measured in the number of 16S rRNA gene copies per swab, as the product of total penile bacterial load and relative (proportional) abundance of each genus in the sample.

Men who acquired HIV had significantly higher abundances of penile anaerobes at study baseline than men who remained HIV negative during the trial, including *Prevotella* (*P* = 0.04), *Dialister* (*P* = 0.01), *Mobiluncus* (*P* = 0.02), *Murdochiella* (*P* = 0.04), and *Peptostreptococcus* (*P* = 0.085) compared to the controls (see Table S1 in the supplemental material). However, total penile bacterial loads were similar in cases and controls at study baseline (*P* = 0.21). Likewise, composition of the penile microbiome at study baseline did not differ significantly between cases and controls (*P* = 0.06 by permutational multivariate analysis of variance [PerMANOVA]) (Fig. S1 and S2). Comparing other risk factors at baseline, we found that higher numbers of nonmarital sexual partners, inconsistent condom use, among other factors were associated with increased HIV acquisition during the trial (Table 1) (2).

10.1128/mBio.00996-17.5 TABLE S1  Penile anaerobe absolute abundance at study baseline in seroconverters (cases) versus men who remained persistently HIV negative (controls). Download TABLE S1, DOCX file, 0.1 MB. Copyright © 2017 Liu et al. 2017 Liu et al. This content is distributed under the terms of the Creative Commons Attribution 4.0 International license.

10.1128/mBio.00996-17.2 FIG S1  Penile anaerobe microbiome at study baseline in seroconverters (cases) versus men who remained persistently HIV negative (controls), visualized by heatmap. Each row represents the results for one man, and cases were labeled by time of detection of HIV seroconversion during the trial. Each column depicts the absolute abundance of a penile coronal sulcus taxon (e.g., *Prevotella*) and can be interpreted using the annotated color-coding key (color bar), which denotes the correlation between each color with its respective log_10_-transformed absolute abundance. Download FIG S1, PDF file, 9.8 MB. Copyright © 2017 Liu et al. 2017 Liu et al. This content is distributed under the terms of the Creative Commons Attribution 4.0 International license.

10.1128/mBio.00996-17.3 FIG S2  Penile anaerobe microbiome at study baseline in seroconverters (cases) versus men who remained persistently HIV negative (controls), visualized by nonmetric multidimensional scaling. Each data point represents the penile microbiome of an individual at trial baseline. The centroids and 95% confidence ellipses for controls and cases by time of detection of HIV seroconversion are as shown. The cases appeared to have more similar penile microbiome at study baseline than controls, and there was a borderline significant difference in penile microbiome composition between cases and controls (*P* = 0.06 by PerMANOVA). Download FIG S2, PDF file, 0.1 MB. Copyright © 2017 Liu et al. 2017 Liu et al. This content is distributed under the terms of the Creative Commons Attribution 4.0 International license.

**TABLE 1:** Study participant characteristics at study baseline

Characteristic	No. (%) of study participants with the indicated characteristic:		Fisher’s *P* value
	Cases (*n* = 46)	Controls (*n* = 136)	
Age (yr)			0.13
15–19	9 (13.0)	41 (30.1)	
20–24	23 (34.8)	36 (26.5)	
25–29	23 (28.3)	24 (17.6)	
30–34	13 (17.4)	25 (18.4)	
35–49	4 (6.5)	10 (7.4)	
Education status			0.55
None	3 (6.5)	7 (5.1)	
Primary	34 (73.9)	91 (66.9)	
Secondary or beyond	9 (19.6)	38 (27.9)	
Marital status			0.06
Never married	20 (43.5)	68 (50.0)	
Currently married	21 (45.6)	65 (47.8)	
Divorced/widowed	5 (10.9)	3 (2.2)	
No. of nonmarital sexual partners in the past yr			0.006
None	10 (21.7)	41 (29.7)	
1	18 (39.1)	47 (34.6)	
2	7 (15.2)	15 (11.0)	
≥3	9 (19.6)	7 (5.1)	
Not sexually active	2 (4.3)	26 (19.1)	
Condom use in the past yr			0.089
Never	16 (34.8)	51 (37.5)	
Sometimes/inconsistent	21 (45.7)	46 (33.8)	
Always	7 (15.2)	15 (11.0)	
Self-reported symptoms of sexually transmitted infection in the past 6 mo			
Genital ulcer disease	2 (4.3)	6 (4.4)	1.00
Urethral discharge	5 (10.9)	4 (2.9)	0.05
Dysuria	4 (8.7)	12 (8.8)	1.00

**FIG 1:**
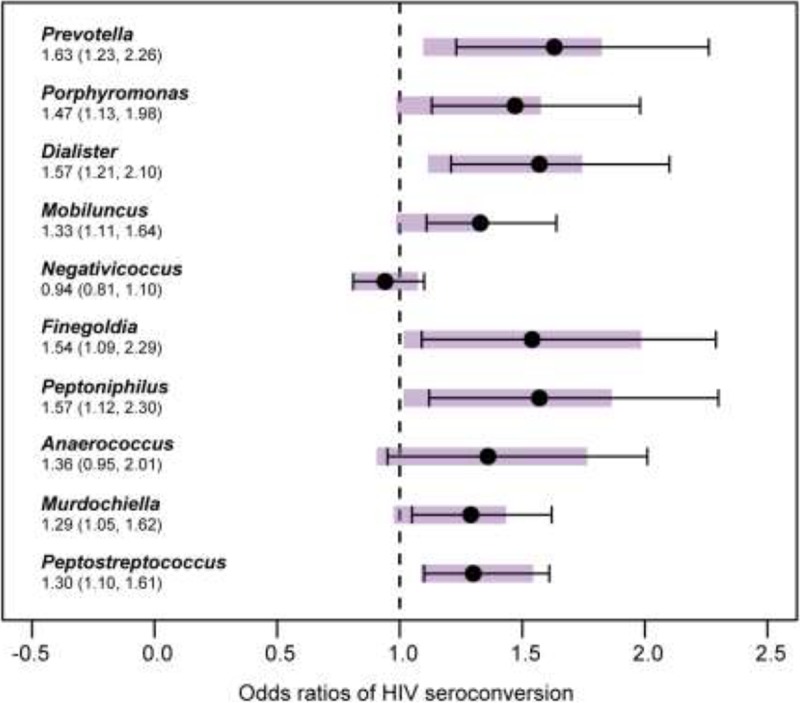
Relationship between the absolute abundances of anaerobic bacteria and risk of HIV seroconversion in the case-control study. At study baseline, each 10-fold increase in the absolute abundance (measured in the log_10_-transformed number of 16S rRNA gene copies per swab) of anaerobic bacteria *Prevotella*, *Dialister*, *Finegoldia*, *Peptoniphilus*, and *Peptostreptococcus* increased the odds of HIV infection. The risk for seroconversion further increased after adjustment for age, marital status, number of extramarital sexual partners, condom use, and genital discharge symptoms. Under each genus, the adjusted odds ratio (i.e., the mean adjusted odds of HIV seroconversion per 10-fold increase in absolute abundance of bacteria) and 95% confidence interval (in parentheses) are shown. Graphically, the 95% confidence interval for the unadjusted odds ratio is shown as purple bar for each genus, and the mean and 95% confidence interval of the adjusted odds ratio is shown as a whisker plot. Detailed results can be found in Table S2 in the supplemental material.

The risk of HIV infection increased with higher penile anaerobic bacterial abundance in regression analyses, which was consistent with the higher baseline mean abundance noted in men who later became infected by HIV (Fig. 1; Table S2). In unadjusted analysis, we found remarkably consistent relationships between anaerobe abundance and HIV seroconversion among 5 of the 10 anaerobic genera at baseline: for each 10-fold increase in the abundance of these organisms, the odds of seroconversion increased by 28 to 40% (Fig. 1, purple bars; Table S2).

10.1128/mBio.00996-17.6 TABLE S2  Odds of HIV seroconversion associated with each 10-fold increase in the abundance of penile anaerobes at study baseline, with and without adjustment for other risk factors. Download TABLE S2, DOCX file, 0.1 MB. Copyright © 2017 Liu et al. 2017 Liu et al. This content is distributed under the terms of the Creative Commons Attribution 4.0 International license.

After adjustment for risk factors, the association between abundance of anaerobic bacteria and the odds of HIV seroconversion actually strengthened (Fig. 1, whisker plots; Table S2), indicating that the increased risk associated with higher densities of anaerobic bacteria was independent of other risk factors. Genera associated with the greatest risk increase in seroconversion with each 10-fold increase in abundance included *Prevotella* (adjusted odds ratio [AdjOR] = 1.63; 95% confidence interval [95% CI], 1.23 to 2.26), followed by *Dialister* (AdjOR = 1.57; 95% CI; 1.21 to 2.10), and six other genera of anaerobic bacteria (*Peptoniphilus*, *Finegoldia*, *Porphyromonas*, *Mobiluncus*, *Peptostreptococcus*, and *Murdochiella*) (Fig. 1; Table S2).

**FIG 2:**
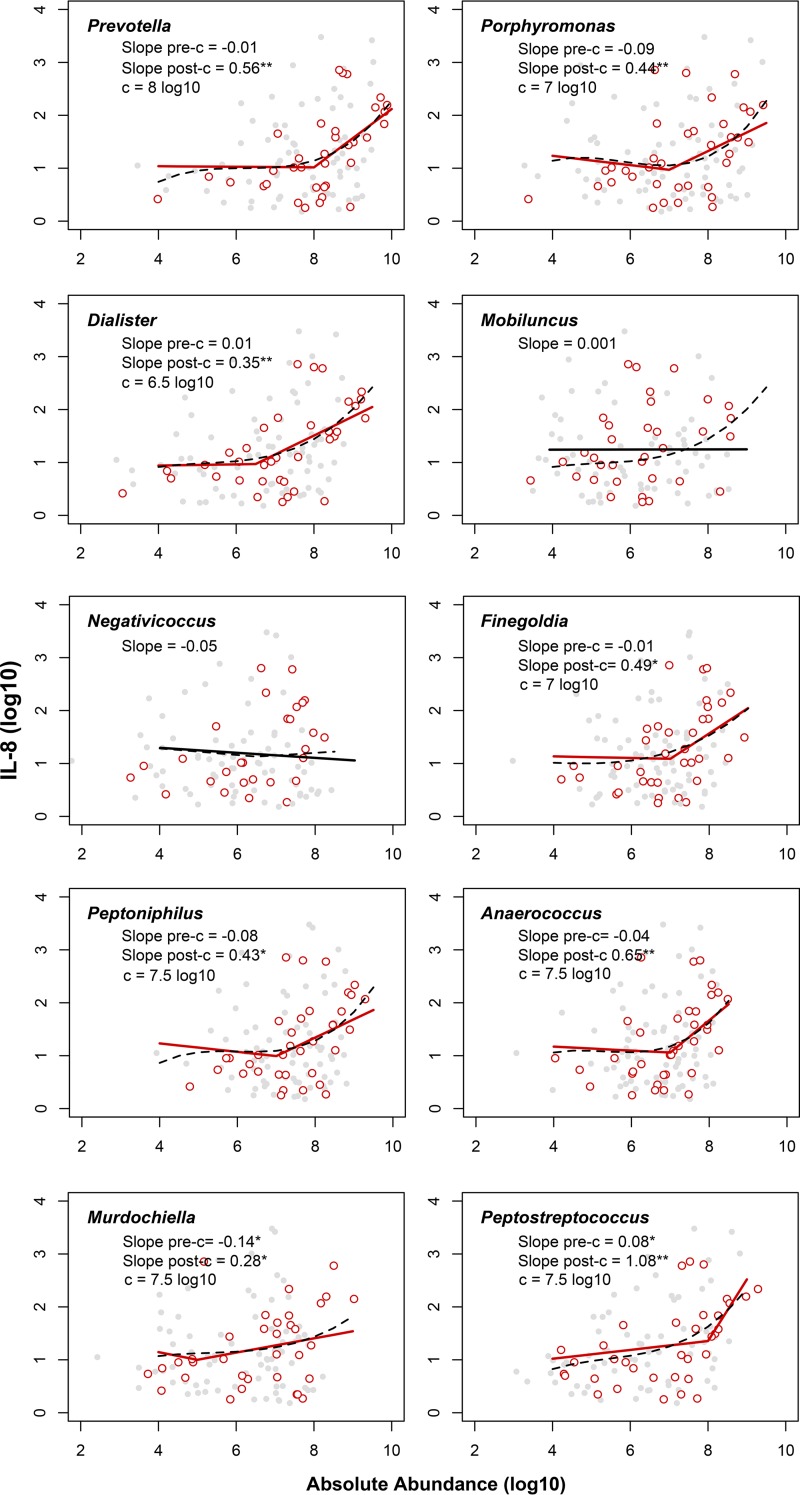
Relationship between absolute abundance of penile anaerobes and concentration of IL-8 in the case-control study. The relationship between anaerobe abundance, measured in the number of 16S rRNA gene copies per swab (log_10_) and IL-8 concentration, measured in picograms per milliliter (log_10_) is similar between men who seroconverted (cases) (red circles) and men who remained HIV negative (controls) (gray circles). The lines describe best-fit models based on AIC selection, which supported spline functions for all anaerobic bacteria except two anaerobic bacteria. Significant relationships (*P* < 0.05) are shown by solid red lines, and nonsignificant relationships are shown by solid black lines. To demonstrate the underlying data trend, polynomial spline models are shown by dashed black lines. Under the name of each genus, c represents the breakpoint used in the piecewise spline model, and pre- and post-c slopes are the slope before and after c, respectively; slopes that are significantly different from zero are indicated by asterisks as follows: *, *P* < 0.05; **, *P* < 0.01. Detailed results can be found in Table S3 in the supplemental material.

Markers of inflammation that have been associated with increased HIV risk (8), particularly penile interleukin-8 (IL-8), increased with the abundance of penile anaerobic bacteria; however, the relationships were not linear (Fig. 2; Table S3). The odds of having any levels of inflammatory cytokines (interleukin 1α [IL-1α], interleukin 8 [IL-8], macrophage chemoattractant 1 [MCP-1], monokine induced by interferon gamma [MIG], macrophage inflammatory protein 3α [MIP-3α], RANTES [*r*egulated on *a*ctivation, *n*ormal *T* cell *e*xpressed and *s*ecreted], and granulocyte-macrophage colony-stimulating factor [GM-CSF]) detected increased significantly with the densities of anaerobic bacteria, where the greatest risk increases were seen in having a higher number, i.e., three or more detected cytokines in the coronal sulcus (Table 2; Fig. S3).

10.1128/mBio.00996-17.7 TABLE S3  Relationship between abundance of anaerobic bacteria and interleukin-8 (IL-8) concentrations with AIC-selected linear regression models with and without linear spline. Download TABLE S3, DOCX file, 0.1 MB. Copyright © 2017 Liu et al. 2017 Liu et al. This content is distributed under the terms of the Creative Commons Attribution 4.0 International license.

10.1128/mBio.00996-17.4 FIG S3  Relationship between the absolute abundance of penile anaerobes and the number of cytokines (IL-1α, IL-8, MCP-1, MIG, MIP-3α, RANTES, and GM-CSF) detected at study baseline. Download FIG S3, PDF file, 0.2 MB. Copyright © 2017 Liu et al. 2017 Liu et al. This content is distributed under the terms of the Creative Commons Attribution 4.0 International license.

**TABLE 2:** Relationship between abundance of penile anaerobes and risk of having greater number of proinflammatory cytokines present at detected levels in the coronal sulcus in the case-control study

Anaerobe	AdjOR (95% CI)^a^		
	1 cytokine vs 0 cytokine	2 cytokines vs 0 cytokine	3 or more cytokines vs 0 cytokine
Gram-negative			
*Prevotella*	1.91 (1.42, 2.57)	1.66 (1.19, 2.33)	4.75 (2.35, 9.62)
*Porphyromonas*	1.78 (1.35, 2.35)	1.39 (1.03, 1.89)	2.03 (1.29, 3.21)
*Dialister*	1.62 (1.27, 2.06)	1.54 (1.15, 2.05)	3.22 (1.86, 5.59)
*Negativicoccus*	1.15 (0.99, 1.35)	0.99 (0.84, 1.18)	1.18 (0.92, 1.52)
*Mobiluncus*	1.45 (1.21, 1.73)	1.16 (0.96, 1.39)	1.28 (1.00, 1.64)
Gram-positive			
*Finegoldia*	1.40 (1.00, 1.95)	1.57 (1.03, 2.39)	4.64 (2.11, 10.21)
*Peptoniphilus*	1.89 (1.34, 2.68)	1.59 (1.08, 2.36)	2.70 (1.47, 4.97)
*Anaerococcus*	1.55 (1.08, 2.21)	1.30 (0.86, 1.95)	2.80 (1.41, 5.58)
*Murdochiella*	1.48 (1.19, 1.83)	1.23 (1.00, 1.53)	1.32 (0.97, 1.78)
*Peptostreptococcus*	1.31 (1.10, 1.54)	1.55 (1.22, 1.97)	3.95 (2.17, 7.20)

a Adjusted odds ratio (95% confidence interval). The odds ratio was adjusted for age, marital status, number of extramarital sexual partners, condom use, and genital discharge symptoms.

IL-8 was the most commonly detected cytokine among those measured. The relationship between many anaerobic genera and IL-8 was best fit by a piecewise linear model, suggesting a threshold effect, where strong associations with IL-8 concentrations in picograms per milliliter (log_10_)—as indicated by the steep slope—were seen only above specific anaerobic bacterial thresholds (i.e., the breakpoint [*c*]) (Fig. 2; Table S3). Above such thresholds, we found that the concentration of IL-8—which can initiate an inflammatory response—was significantly correlated with the absolute abundances of penile anaerobes associated with higher risk of seroconversion, such as *Prevotella* (slope after the breakpoint [*c*] used in the piecewise spline model [slope_post-*c*_] = 0.56; *c* = 8 log_10_ copies of 16S rRNA gene per swab; *r*^*2*^ = 0.14), *Dialister* (slope_post-*c*_ = 0.35; *c* = 6.5 log_10_ copies of 16S rRNA gene per swab; *r*^*2*^ = 0.15), and *Peptostreptococcus* (slope_post-*c*_ = 1.08; *c* = 6.5 log_10_ copies of 16S rRNA gene per swab; *r*^*2*^ = 0.19) (Fig. 2). In contrast, *Negativicoccus*, which was unrelated to HIV seroconversion, was not associated with concentrations of IL-8 at any level of abundance (slope = −0.05; *r*^*2*^ = 0.03) (Fig. 2; Table S3).

## DISCUSSION

High densities of anaerobic bacteria on the penis appeared to increase the risk of HIV acquisition in men, a novel risk factor that operated independently of other known risk factors that we measured. We found that as the absolute abundance of anaerobes increased, the concentrations of chemokines in the penile coronal sulcus increased as well, suggesting a plausible mechanism whereby anaerobic dysbiosis influences HIV infection risk.

This study focused on the risk of HIV seroconversion associated with penile anaerobe abundance using samples from uncircumcised men as the trial baseline. As a result, our study’s estimates are likely conservative, given that the potential shift in the penile microbiome from the study baseline to time of seroconversion is expected to attenuate our findings. By using baseline samples, we ensured that the differences in microbiome composition preceded HIV infection, which can have a major effect on the microbiome. The temporal shift in the penile microbiome is expected to be limited based on our earlier study of the penile microbiome in HIV-uninfected uncircumcised men (17).

We previously found in the same cohort of men that greater HIV risk was associated with increased IL-8 and other chemokines in the coronal sulcus (8), and the current work strongly suggests that the penile microbiome affects chemokine levels in the coronal sulcus. Higher densities of anaerobic bacteria could induce the production of the chemokine interleukin-8 (18), which primarily recruits neutrophils (19–21). Neutrophils can be further activated by bacterial antigens to produce MIP-3α and MCP-1, chemokines that recruit Th_17_ helper T cells (22–24), a T-cell subset especially susceptible to HIV infection (25–28). Thus, the response of the immune system to shifts in the penile microbiome may facilitate productive infection by HIV. In other words, the genital microbiome may influence seroconversion risk by modulating the very target of HIV infection—the human immune system.

Anaerobic dysbiosis may be a general mechanism affecting the risk of HIV acquisition in both men and women. A similar anaerobic dysbiosis occurs in women with bacterial vaginosis (29), which also increases their risk of HIV infection (15, 30–33). In men, this anaerobic dysbiosis largely resolves in response to circumcision (17), an intervention that reduces the risk of HIV infection (2–4). Furthermore, these anaerobic bacteria are shared by sexual partners, indicating that the dysbiosis itself is sexually transmissible (34). Our findings explain these independent lines of evidence and implicate sexually transmitted anaerobic dysbiosis in the genital microbiome as a risk factor for HIV infection that is not only common to both men and women but also sexually transmissible between them.

Our findings support the potential for novel HIV prevention strategies aimed at reducing the abundance of high-risk genital anaerobes. While hygiene improvements such as genital washing have not been effective in reducing HIV risk (35), narrow-spectrum antimicrobials, such as bacteriocins, or phage therapies that eliminate specific anaerobes (36, 37), or pre- and probiotic strategies may reduce colonization by high-risk genital anaerobes. Such methods may be used independently or as adjuvants to other interventions to further reduce HIV risk.

## MATERIALS AND METHODS

### Study design and sample collection.

We conducted a randomized trial of male circumcision for HIV prevention in 2004 to 2006 (2). In this study, HIV-uninfected, uncircumcised men 15 to 49 years old were randomized to either immediate circumcision or circumcision delayed for 24 months as described previously (2, 38). At enrollment, all consenting men provided interview information on sociodemographic, behavioral, and health characteristics and underwent a physical examination. Study participants were provided access to regular reproductive health services and monitored 6, 12, and 24 months later for up to 24 months to assess HIV status and sexually transmitted infection (STI) acquisition, as described in detail elsewhere (2). Microbiome and cytokine data were drawn from the baseline visit.

At each visit, clinical officers collected penile swabs using premoistened Dacron swabs and rotated twice around the full circumference of the penis at the coronal sulcus, the junction between the glans and the shaft of the penis. The swabs were immediately placed in 1-ml Digene specimen transport medium (Roche Diagnostics, Indianapolis, IN) at 4°C for less than 4 h and then vortexed, aliquoted, and stored at −80°C until analysis. The study was approved by four institutional review boards (IRBs): the Science and Ethics Committee of the Uganda Virus Research Institute (Entebbe, Uganda), the HIV subcommittee of the National Council for Science and Technology (Kampala, Uganda), the Committee for Human Research at Johns Hopkins University’s Bloomberg School of Public Health (Baltimore, MD), and the Western Institutional Review Board, the IRB of record for the Translational Genomics Research Institute (Olympia, WA).

The current case-control study included only trial participants randomized to the delayed circumcision arm of the trial, who remained uncircumcised for the duration of the trial, as medical male circumcision can drastically alter the penile microbiome (17). Cases were defined as participants who seroconverted during the 2-year study period, and the controls were participants who remained HIV uninfected during the 2-year study period. Each case was matched to three controls selected at random.

### Penile microbiome characterization.

DNA isolation and purification were performed using the collected coronal sulcus swabs as previously described (38). Briefly, 500 μl of 1 ml of Digene specimen transport medium was used in DNA extraction with the QIAamp DNA blood MDx kit (Qiagen Inc., Valencia, CA, USA) per the manufacturer’s instructions. The purified DNA was eluted into 100 μl of Tris-EDTA (TE) buffer and stored at −80°C until quantitative PCR (qPCR) and sequencing analyses.

The total penile bacterial load from each swab was measured using a 16S rRNA gene-based broad-coverage qPCR targeting the V3-V4 region as described previously (34, 39) using 1 µl of purified DNA in 10-µl reaction mixture volumes on the Applied Biosystems 7900HT fast real-time PCR system (Thermo Fisher Scientific, Foster City, CA, USA).

The relative abundances of penile bacteria in each coronal sulcus swab were characterized by sequencing the 16S rRNA gene V3-V4 region using a two-step PCR protocol validated for samples with low biomass. Briefly, the first PCR step incorporates Illumina MiSeq 5′ sequencing primer (CS1/CS2), a heterogeneity spacer, and 16S rRNA gene primers, and the second PCR step incorporates Illumina MiSeq 3′ Flowcell Linker sequence, a 6-bp sample-specific index, and complementary sequences to the MiSeq 5′ sequencing primer (CS1/CS2) from the first amplification. Additional details on amplicon sequencing analysis can be found in Text S1 in the supplemental material. The resultant amplicon library was sequenced on the Illumina MiSeq platform using 600 cycles to produce 300 bp paired-end reads per the manufacturer’s instructions (Illumina Inc., San Diego, CA).

10.1128/mBio.00996-17.1 TEXT S1  Supplemental methods. Download TEXT S1, DOCX file, 0.1 MB. Copyright © 2017 Liu et al. 2017 Liu et al. This content is distributed under the terms of the Creative Commons Attribution 4.0 International license.

The resultant paired-end reads were processed as follows. First, the reads were trimmed to retain bases with high quality scores. The quality-checked reads were then assembled to generate a single stitched read with dual barcodes; the single stitched reads were then assigned to the original samples and underwent additional quality checks and trimming. The resultant binned and trimmed sequences were dereplicated at a similarity level of 97% using USEARCH (version 5.2.32), and chimeric sequences were removed using UCHIME (version 5.1) (40). Following this, the resultant sequences were classified at each taxonomic level at ≥97% bootstrap confidence level using a web service for the Naïve Bayesian Classifier (v.2.10) (41). Classification results for each sample are enumerated to generate an abundance matrix for analysis. Additional details on bioinformatic analysis can be found in Text S1.

### Cytokine measurement.

An electrochemiluminescence detection system using a custom human Ultra-Sensitive kit from Meso Scale Discovery (Rockville, MD) was used to assay cytokines in coronal sulcus swabs. All cytokines examined were detected in coronal sulcus swabs; however, as cytokine levels other than IL-8 were generally low, we analyzed detection of the seven cytokines measured as present or absent based on the lower limit of quantification (LLoQ) for each cytokine. IL-8 was detected in 60% of coronal sulcus swabs (range, >1.5 to 7,405.7 pg/ml) and also analyzed as a continuous variable. The LLoQs used in this study were as follows: 0.6 pg/ml for IL-1α, 1.5 pg/ml for IL-8, 0.6 pg/ml for MCP-1, 0.3 pg/ml for MIG, 3.0 pg/ml for MIP-3α, 0.6 pg/ml for RANTES, and 0.3 pg/ml for GM-CSF.

### Absolute abundance estimation.

The absolute abundance of each of the 10 anaerobic genera shown to be reduced by male circumcision was calculated as absolute abundance = total penile bacterial load (measured as the total number of copies of 16S rRNA gene per swab by broad-coverage real-time PCR) × relative abundance of a specific anaerobic genus (measured as the number of 16S rRNA gene sequences assigned to a genus in a given sample divided by the total number of 16S rRNA sequences obtained for that sample from sequencing). Log_10_-transformed absolute abundance was used in the linear and logistic models.

### Statistical analysis.

We compared the demographic and behavioral characteristics of cases and controls at trial enrollment by Fisher exact test to inform adjusted models. The total penile bacterial load and absolute abundance of 10 penile anaerobes of cases and controls were compared by Kolmogorov-Smirnov test. Penile microbiome composition of cases and controls were compared by permutational analysis of variance using absolute abundance data in Euclidean distance and 999 permutations. The relationships between the abundance of penile anaerobes and risk of HIV seroconversion were assessed using univariate and multivariate logistic regression models, regressing participant status (case/control) on the log_10_-transformed penile anaerobe abundance, performed separately for each genus. The multivariate models were adjusted for age, marital status, number of extramarital sexual partners, condom use, and genital discharge symptoms, all of which are previously established epidemiologic risk factors (2).

The odds of having 1, 2, or ≥3 cytokines detected compared to no cytokines detected associated with each 10-fold increase in bacterial abundance were determined using multinomial logistic regression models. We also examined the relationship between foreskin IL-8 concentrations and abundance of penile anaerobes determined using piecewise linear regression, where a single breakpoint (*c*) for the spline model was empirically selected based on *r*^*2*^. Slope estimates (slope_pre-*c*_ and slope_post-*c*_) representing the slope before and after the breakpoint and their respective *P* values, as well as Akaike information criterion (AIC) scores from comparisons of the linear model to the piecewise linear model were presented. The underlying data trend characterized using a nonparametric b-spline function was plotted together with the best-fitting model. All statistical analyses were performed in R 3.2.4.
