# Supplementary material for: Penile Anaerobic Dysbiosis as a Risk Factor for HIV Infection
Source: mBio. 2017 Jul 25;8(4):e00996-17. doi: 10.1128/mBio.00996-17 (PMC5527312; doi:10.1128/mBio.00996-17)
Supplement: TABLE S1 [file mbo004173393st1.docx]

SUPPLEMENTARY RESULTS

Table S1. Penile anaerobe absolute abundance at study baseline in seroconverters (cases) versus men who remained persistently HIV-negative (controls)

|  | Cases (n = 46) | Controls (n = 136) | *p-value* |
| --- | --- | --- | --- |
|  | Median (*Q1-Q3*) | |  |
| Total penile bacterial load | 4.05 x 10^8^  (*3.36 x 10^7^-2.35 x 10^9^*) | 2.06 x 10^8^ (*1.87 x 10^7^-1.49 x 10^9^*) | *p =0.21* |
| Gram-negative |  |  |  |
| *Prevotella* | 1.47 x 10^8^  (*9.26 x 10^6^-5.87 x 10^8^*) | 1.87 x 10^7^ (*8.60 x 10^5^-3.10 x 10^8^*) | *p =0.04* |
| *Porphyromonas* | 1.21 x 10^7^  (*1.60 x 10^6^-1.29 x 10^8^*) | 5.88 x 10^6^ *(1.97 x 10^5^-8.31 x 10^7^*) | *p =0.11* |
| *Dialister* | 7.04 x 10^6^  (*9.49 x 10^5^-9.59 x 10^7^*) | 1.36 x 10^6^ (*6.08 x 10^4^-3.55 x 10^7^*) | *p =0.01* |
| *Negativicoccus* | 4.91 x 10^5^  (*2.32 x 10^3^-1.93 x 10^7^*) | 4.29 x 10^5^  (*1.37 x 10^4^-7.21 x 10^6^*) | *p =0.37* |
| *Mobiluncus* | 6.79 x 10^5^  (*1.17 x 10^5^-3.63 x 10^6^*) | 1.61 x 10^5^ (*5.23 x 10^3^-9.49 x 10^6^*) | *p =0.02* |
| Gram-positive |  |  |  |
| *Finegoldia* | 6.62 x 10^6^  (*1.82 x 10^6^-4.75 x 10^7^*) | 3.47 x 10^6^ (*5.42 x 10^5^-1.50 x 10^7^*) | *p =0.06* |
| *Peptoniphilus* | 2.04 x 10^7^  (*3.69 x 10^6^-1.75 x 10^8^*) | 1.59 x 10^7^ (*1.59 x 10^6^-7.60 x 10^7^*) | *p =0.20* |
| *Anaerococcus* | 7.24 x 10^6^  (*1.06 x 10^6^-4.04 x 10^7^*) | 4.14 x 10^6^ (*7.57 x 10^5^-1.80 x 10^7^*) | *p =0.25* |
| *Murdochiella* | 1.71 x 10^6^  (*1.71 x 10^5^-3.11 x 10^7^*) | 6.91 x 10^5^  (*2.42 x 10^4^-1.00 x 10^7^*) | *p =0.04* |
| *Peptostreptococcus* | 2.58 x 10^6^  (*5.74 x 10^4^-5.18 x 10^7^*) | 1.48 x 10^5^ (*8.03 x 10^3^-3.43 x 10^6^*) | *p =0.008* |
