# Supplementary figures and images for: Penile Anaerobic Dysbiosis as a Risk Factor for HIV Infection
Source: mBio. 2017 Jul 25;8(4):e00996-17. doi: 10.1128/mBio.00996-17 (PMC5527312; doi:10.1128/mBio.00996-17)

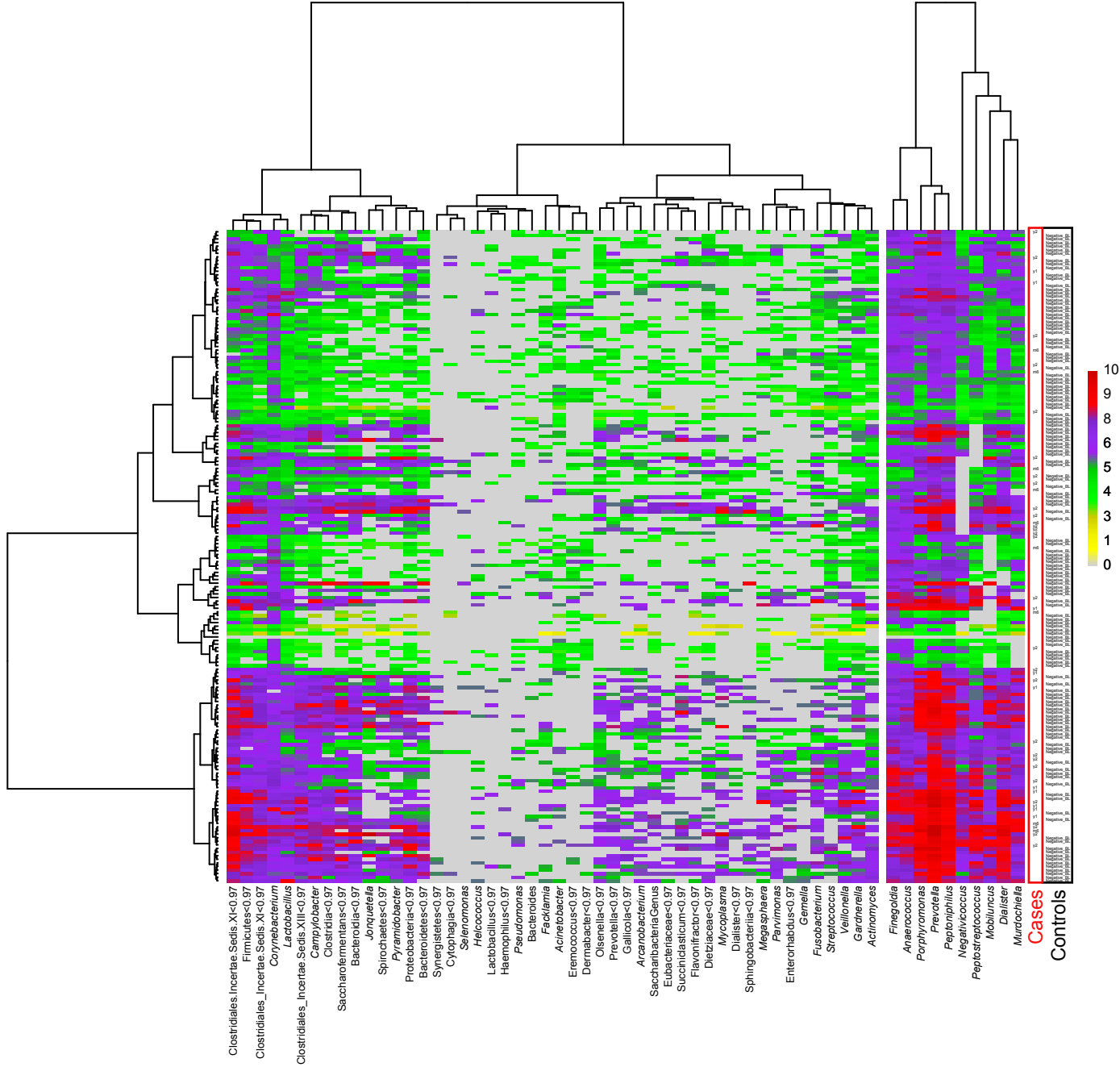

Supplement: FIG S1 [file mbo004173393sf1.pdf]

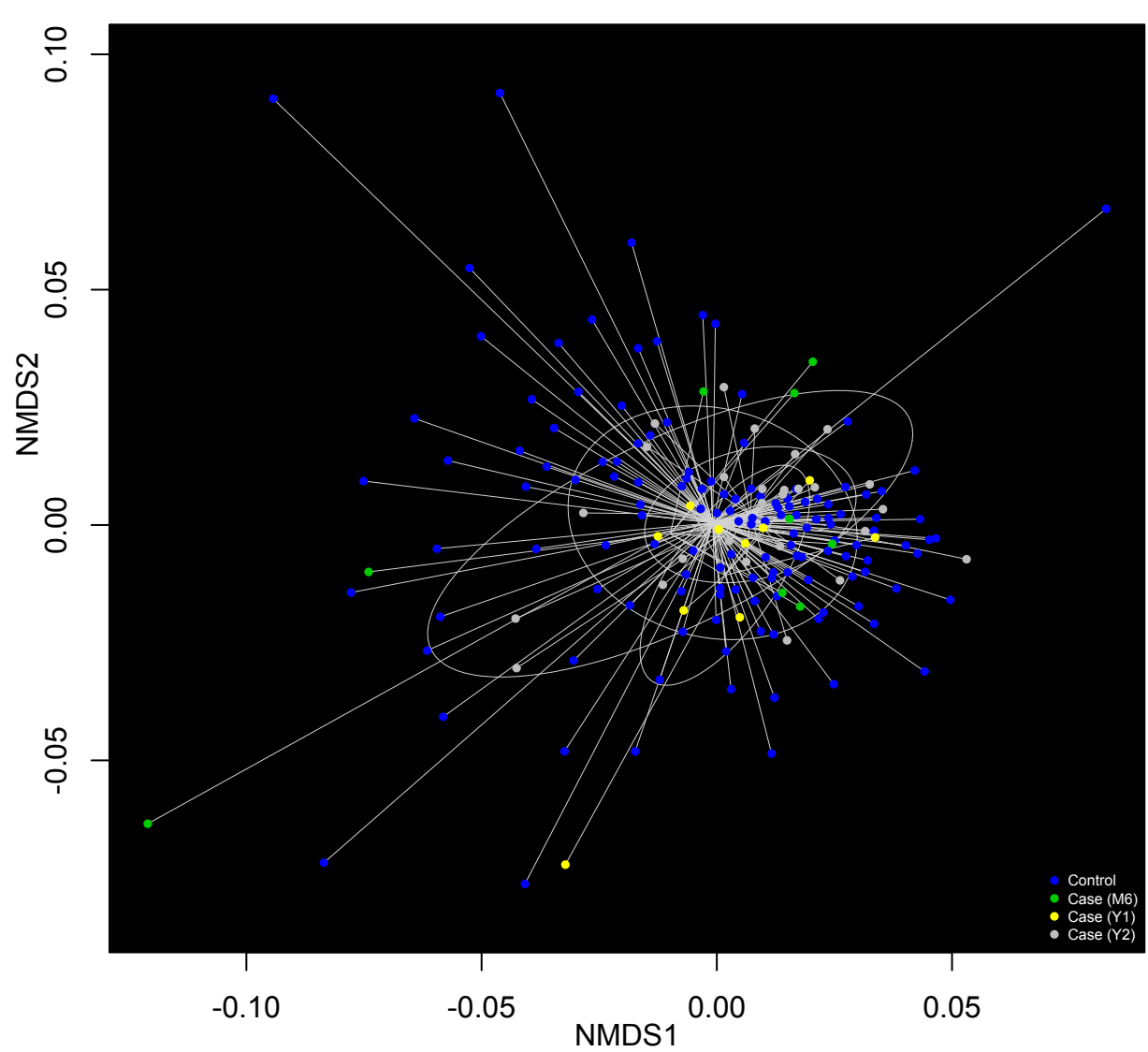

Supplement: FIG S2 [file mbo004173393sf2.pdf]

Absolute Abundance (log10)

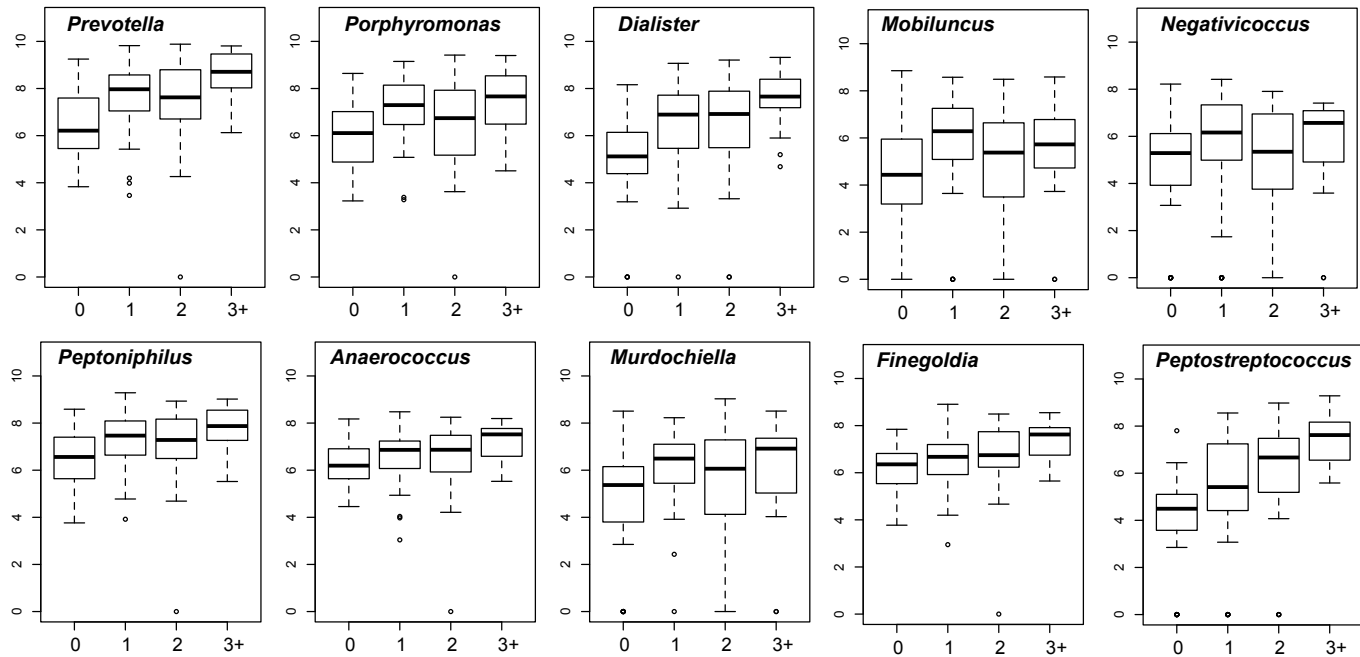

Number of detectable cytokines

Supplement: FIG S3 [file mbo004173393sf3.pdf]
