# Supplementary material for: Penile Anaerobic Dysbiosis as a Risk Factor for HIV Infection
Source: mBio. 2017 Jul 25;8(4):e00996-17. doi: 10.1128/mBio.00996-17 (PMC5527312; doi:10.1128/mBio.00996-17)
Supplement: TABLE S2 [file mbo004173393st2.docx]

Table S2. Odds of HIV seroconversion associated with each 10-fold increase in abundance of penile anaerobes at study baseline, with and without adjustment for other risk factors

|  |  |  |  |  |  |
| --- | --- | --- | --- | --- | --- |
|  | Unadjusted | | Adjusted^§^ | |  |
|  | Odds Ratio | 95% CI | Odds Ratio | 95% CI |  |
| Gram-Negative |  |  |  |  |  |
| *Prevotella* | 1.40* | (1.10, 1.82) | 1.63* | (1.23, 2.26) |  |
| *Porphyromonas* | 1.24 | (0.99, 1.57) | 1.47* | (1.13, 1.98) |  |
| *Dialister* | 1.37* | (1.12, 1.74) | 1.57* | (1.21, 2.10) |  |
| *Negativicoccus* | 0.93 | (0.82,1.07) | 0.94 | (0.81, 1.10) |  |
| *Mobiluncus* | 1.14 | (0.99, 1.34) | 1.33* | (1.11, 1.64) |  |
| Gram-Positive |  |  |  |  |  |
| *Finegoldia* | 1.40* | (1.02, 1.98) | 1.54* | (1.09, 2.29) |  |
| *Peptoniphilus* | 1.36* | (1.02, 1.86) | 1.57* | (1.12, 2.30) |  |
| *Anaerococcus* | 1.24 | (0.91, 1.76) | 1.36 | (0.95, 2.01) |  |
| *Murdochiella* | 1.17 | (0.98, 1.43) | 1.29* | (1.05, 1.62) |  |
| *Peptostreptococcus* | 1.28* | (1.09, 1.54) | 1.30* | (1.10, 1.61) |  |
| *Statistically significant  §Adjusted for age, marital status, number of extramarital sexual partners, condom use, and genital discharge symptoms | | | | | |
