# Supplementary material for: Penile Anaerobic Dysbiosis as a Risk Factor for HIV Infection
Source: mBio. 2017 Jul 25;8(4):e00996-17. doi: 10.1128/mBio.00996-17 (PMC5527312; doi:10.1128/mBio.00996-17)
Supplement: TABLE S3 [file mbo004173393st3.docx]

Table S3. Relationship between abundance of anaerobic bacteria and interleukin-8 (IL-8) concentrations with AIC-selected linear regression models with and without linear spline

|  | Linear Regression | | | | | |
| --- | --- | --- | --- | --- | --- | --- |
|  | Effect before *c^*^* | | Effect after *c^*^* | | | *r^2^* |
|  | Slope_pre-_*_c_* | *p*-value | *c** | Slope_post-_*_c_* | *p*-value |  |
| *Prevotella* | -0.01 | 0.930 | 8.00 | 0.56 | 0.002 | *0.14* |
| *Porphyromonas* | -0.09 | 0.240 | 7.00 | 0.44 | 0.007 | *0.09* |
| *Dialister* | 0.01 | 0.840 | 6.50 | 0.35 | 0.009 | *0.15* |
| *Mobiluncus* | <-0.01 | 0.972 |  |  |  | *<0.01* |
| *Negativicoccus* | -0.05 | 0.084 |  |  |  | *0.03* |
| *Finegoldia* | -0.01 | 0.850 | 7.00 | 0.49 | 0.020 | *0.08* |
| *Peptoniphilus* | -0.08 | 0.390 | 7.50 | 0.43 | 0.020 | *0.07* |
| *Anaerococcus* | -0.04 | 0.630 | 7.50 | 0.65 | 0.008 | *0.08* |
| *Murdochiella* | -0.14 | 0.040 | 7.50 | 0.28 | 0.020 | *0.05* |
| *Peptostreptococcus* | 0.08 | 0.005 | 7.50 | 1.08 | 0.003 | *0.19* |

**c* represents the breakpoint used in the piecewise linear spline model
