# Supplementary material for: Penile Anaerobic Dysbiosis as a Risk Factor for HIV Infection
Source: mBio. 2017 Jul 25;8(4):e00996-17. doi: 10.1128/mBio.00996-17 (PMC5527312; doi:10.1128/mBio.00996-17)
Supplement: TEXT S1 [file mbo004173393s1.docx]

SUPPLEMENTARY METHODS

16S rRNA gene-based amplicon sequencing

Microbiome from each coronal sulcus swab was characterized by sequencing the 16S rRNA gene V3-V4 region using a two-step PCR protocol validated for samples with low biomass. Briefly, the first PCR step included the following: 1X Phusion Taq Master Mix (Thermo Fisher Scientific, Halethrope, MD, USA), 3% DMSO, and 0.4 μM each of fusion primers 319F (5’- *GTGACTGGAGTTCAGACGTGT-GCTCTTCCGATCT*-Heterogeneity Spacer-ACTCCTACGGGAGGCAGCAG -3’) and 806R (5’- *ACACTCTTTCCCTACACGACGCT-CTTCCGATCT*-Heterogeneity Spacer-GGACTACHVGGG-TWTCTAAT -3’) primers, where the italicized sequences are the Illumina MiSeq 5' Sequencing Primer (CS1/CS2), and the bolded sequences sequences denote bacterial 16S rRNA gene primer sequences. Purified genomic DNA samples (1pg-10ng) were amplified using the following cycling conditions: an initial denaturation at 94.0°C for 3 min, 20 cycles of denaturation at 94.0°C for 30 s, annealing at 58.0°C for 30 s, and elongation at 72.0°C for 1 min, and a final elongation step at 72.0°C for 7 min.

The second PCR step used another set of fusion primers Forward (5’-*CAAGCAGAAGACGGC-ATACGAGAT*nnnnnnCACTGACCTCAAGTCTGCACACGAGAAGGCTAGA-3’) and Reverse (*AATGATACGGCGACCACCGAGATCT*nnnnnnTGTGAGAAAGGGATGTGCTGCGAGAAGGCTAGT-3’), where the italicized sequences denotes Illumina MiSeq 3' Flowcell Linker sequence, the underlined portion denotes a 6-bp sample-specific index, and the bolded sequences are complementary sequences to MiSeq 5' Sequencing Primer (CS1/CS2) from the first amplification. One μL of 1:20 dilution of amplicons from the first PCR was used in this second PCR step with 0.4 μM each of primer the 1X Phusion Taq Master Mix, and 3% DMSO and was amplified with the following cycling conditions: an initial denaturation at 94°C for 30 s, 10 cycles consisting of denaturation at 94.0°C for 30 s, annealing at 58.0°C for 30 s, and elongation at 72.0°C for 60 s, followed by a final elongation step at 72.0°C for 5 min. Resultant amplicons were purified with the SequalPrep kit (Thermo Fisher Scientific, Halethrope, MD, USA) and sequenced on the Illumina MiSeq platform using 600 cycles producing 2 x 300 bp paired end reads as per manufacturer’s instructions (Illumina Inc., San Diego, CA, USA).

Sequencing data processing and taxonomic classification

A. Raw sequence processing and stitching of paired-end reads. The resultantant 2 x 300 bp paired end reads were processed with Trimmomatic (version 0.35) {Bolger, 2014 #422} to remove the first and last 3 bases if phred score < 3 and truncate reads when average quality score in a 4-bp sliding window is <15. Paired-end reads were assembled using FLASH {Magoc, 2011 #423} with error correction on the ~90 bp overlapping region.

B. Sequence binning, barcode/primer removal, quality trimming, and chimeric sequence removal.

With our sequencing protocol, one barcode was present on each paired end read; this dual-barcoding scheme was used to identify and bin sequences from each samples using a QIIME utility (version 1.8.0) {Caporaso, 2010 #152}. Additional quality trimming was performed in QIIME using the following criteria: 1) truncation when average quality score of next 3-bp is <15, 2) trimming of ambiguous bases, and 3) final sequence length of ≥230-bp. Next, sequences from barcodes, heterogeneity spacers, and primers were trimmed. Clustering was performed at 97% similarity level using USEARCH (version 5.2.32) {Edgar, 2010 #150} and *de novo* chimera detection was conducted in UCHIME (version 5.1) {Edgar, 2011 #151}.

C. Taxonomic Classification. The resultant demultiplexed and quality-checked 16S rRNA gene sequences were classified at each taxonomic level (i.e., phylum, class, order, family, genus) at five bootstrap confidence levels: ≥80%, ≥90%, ≥95%, ≥97% and ≥99% using a web service for the Naïve Bayesian Classifier (v.2.10){Cole, 2009 #81;Wang, 2007 #153}. The taxonomic classifications assigned to the sequences through the RDP Classifier fall into the modern high-order bacterial proposed by Garrity *et. al* {Garrity, 2007 #154}. Classification results for each sample are enumerated to generate an abundance-based matrix for data analysis.

Heatmap and non-metric multidimensional scaling (nMDS) visualization

1. Heatmap visualization
   1. Penile microbiome composition (using log_10_-transformed absolute abundance) was visualized by heatmap for the 10 anaerobes of interest and the remaining penile bacterial taxa. Hierarchal clustering of the individuals’ penile microbiome (*by row*) and of abundance of each penile bacterial taxon (*by column*) was performed by Ward linkage using the squared dissimilarity matrix in Euclidean distance. All analyses were performed in R 3.2.4.
2. nMDS
   1. Penile microbiome composition (using log_10_-transformed absolute abundance) was visualized by non-metric multidimensional scaling to reduce the multidimensional microbiome data into a two-dimensional representation. Dissimilarity matrix generated using log_10_-transformed absolute abundance in Euclidean distance was analyzed using the *metaMDS* function with 100 iterations in *vegan*.
